# Sense of coherence or self-efficacy as predictors of health-related quality of life in sickle cell disease patients

**DOI:** 10.1007/s00277-022-05007-2

**Published:** 2022-11-04

**Authors:** Mehrnaz Ahmadi, Samira Beiranvand, Saeed Poormansouri, Mahsa Matbouei, Camelia Rohani

**Affiliations:** 1 Medical and Surgical Nursing Department, School of Nursing and Midwifery, Nursing Care Research Center in Chronic Diseases, Ahvaz Jundishapur University of Medical Sciences, Ahvaz, Iran; 2 Treatment Deputy, Ahvaz Jundishapur University of Medical Sciences, Ahvaz, Iran; 3 Community Health Nursing Department, School of Nursing & Midwifery, Shahid Beheshti University of Medical Sciences, Tehran, Iran; 4 School of Nursing & Midwifery, Semnan University of Medical Sciences, Semnan, Iran; 5 Present Address: Department of Health Care Sciences, Palliative Care Center, Marie Cederschiöld Högskola, Campus Ersta, Stigbergsgatan 30, Box 11189, SE-100 61 Stockholm, Sweden

**Keywords:** Sickle cell disease, Health-related quality of life, Sense of coherence, Self-efficacy

## Abstract

Patients with sickle cell disease (SCD) suffer from impaired health-related quality of life (HRQoL). This study aimed to determine the level of HRQoL, sense of coherence (SOC), and self-efficacy (SE) in a sample of SCD patients, and to explore predictors of their physical and mental HRQoL. A cross-sectional descriptive study was conducted on 83 SCD patients of one university hospital. The data of the study was collected through Persian versions of the Short-Form Health Survey SF-36 (RAND 36-item), the Sense of Coherence Scale (SOC-13), and the Sickle Cell Self-Efficacy Scale (SCSES). The mean age of the patients was 26.34 ± 8.19 years old. Patients’ mean scores for the Physical Component Summary (PCS), Mental Component Summary (MCS), SOC, and SCSES were 40.57 ± 17.18 (range: 0–100), 50.44 ± 17.95 (range: 0–100), 52.40 ± 15.35 (range: 13–91), 26.40 ± 6.96 (range: 9–45), respectively. Regression models showed that the level of the patients’ SOC, was the main predictor of the MCS (*β* = 0.37, *p* < 0.001). However, the level of the patients’ SE was the main predictor of the PCS (*β* = 0.30, *p* = 0.004). Also, “blood transfusion history” in patients was a common predictor for both the PCS (*β* =  − 0.28, *p* = 0.008) and the MCS (*β* =  − 0.29, *p* = 0.003). These results can assist nurses and clinicians to plan clinical interventions for SCD patients by focusing on increasing the level of the SOC and SE and improving SCD patients’ HRQoL. Furthermore, measuring the level of the SOC and self-efficacy as screening tests are useful to find patients with a greater risk of impaired HRQoL.

## Introduction

Sickle cell disease (SCD) is an inherited hemoglobin disorder that has affected on the health of millions of individuals throughout the world [1]. It has been estimated that about 300 million people worldwide carry the sickle cell trait [2]. SCD is particularly common among sub-Saharan Africa, Spanish-speaking regions, Saudi Arabia, India, and Mediterranean countries [3]. The Eastern Mediterranean is a bridge that links Asia, Africa, and Europe together and Iran is in this region. Statistics show that the prevalence of sickle cell thalassemia (SCT) and SCD is about 1.43% and 0.1%, respectively in the South of Iran. The highest prevalence of the disease has been reported in the South of Iran (Khuzestan Province-City of Ahwaz) due to the Arab Indian haplotype [4].

SCD can cause severe and life-threatening complications such as blindness, acute chest syndrome, gallstones, stroke, renal failure, spleen detachment, priapism, bacterial sepsis, and chronic organ damage [5, 6]. However, an unpredictable crisis of pain is one of the most common complications reported by patients. This is the main reason for frequent visits to the emergency department and hospital to receive medical care [7, 8].

SCD patients experienced high mortality at a young age in the past. But today, the average lifespan of these patients has increased up to the age 50 by using prophylactic antibiotics (such as penicillin), vaccination, and severity-reducing treatments (such as hydroxyurea) [5, 9]. Despite the increase in lifespan, there are defects in some of their health domains. Unpredictable pain, frequent visits to the emergency units and hospitalization, and lack of occupation have led to low self-esteem, feelings of hopelessness, depression, anxiety, and stress in these patients [1, 5, 10, 11].

Obviously, such issues can lead to complications in the psychosocial functioning of SCD patients and reduce their quality of life (QoL) compared to healthy population [12]. Many studies have reported poor QoL, low self-efficacy (SE), and decreased adaptation skills in SCD patients [13–16]. Various factors influence the fluctuations of the patients’ health-related quality of life (HRQoL). Studies have shown that SCD patient’s QoL significantly is improved with their pain relief [1, 7, 12]. In an earlier study, poor QoL of SCD patients attributed essentially to their low economic and social status [17]. Sehlo and colleagues [18] found a direct relationship among SCD patients’ QoL, their social support and self-confidence, and a reverse relationship between their QoL and depression. Furthermore, a relationship was found between the QoL of adolescents with SCD and their adaptive behaviors. With increasing their adaptation, their QoL improved [19]. In our study, we measured the physical and mental HRQoL of SCD patients, as these variables reflect a comprehensive perspective of patient’s HRQoL in various aspects of life that are influenced by the disease [17]. SE is known as one of the factors that improve SCD patients’ QoL. Patients with high QoL had a high level of SE [20]. According to Bandura’s social cognitive theory, SE is a person's belief in his/her ability to do tasks in relation to the symptoms and disease management. It is the basis of motivation for behavioral change and can lead him/her to overcome obstacles [21]. Few studies on the SE of SCD patients showed that the level of the SE is a determining factor in adaptation with the chronic condition. Also, the level of the patients’ SE can influence the prediction of their disease [14]. Patients with higher levels of the SE reported slighter [22] and fewer disease symptoms [15]. Furthermore, SCD patients with lower levels of the SE expended more health care facilities compared to their counterparts with higher levels of the SE [20].

SOC is an intrinsic factor affecting patients’ QoL. Higher QoL and better adaptation to the disease have been reported in patients with high SOC [23]. The SOC is a concept that is used to describe how a person adapt with crises and challenges in his/her life. It was first proposed by Antonovsky in a comprehensive model of the origin of health [24]. He defines the concept of SOC as an orientation to life with three components of comprehensibility (cognitive component), manageability (behavioral component), and meaningfulness (motivational component), which indicates a person’s capacity for effective management of stressful situations [25]. Higher SOC, have better adapted to the chronic disease over time [26]. In earlier studies, a positive correlation has been also reported between the SE and the SOC [27], and a reverse correlation between self-reported pain and the level of the SOC in patients [16].

After a comprehensive literature review, based on our knowledge, there is no study on the SOC of SCD patients. Considering the prevalence of the SCD in our context (Khuzestan Province-Iran) and exploring complex relationships of the SOC and the SE with HRQoL, we decided to conduct this study. Thus, this study aimed to determine the level of physical and mental HRQoL, SOC, and SE in a sample of SCD patients and to explore predictors of their physical and mental HRQoL.

## Methods

### Study design

This is a cross-sectional study with a descriptive design. It was approved by the Research Ethics Committee of two universities: Shahid Beheshti University of Medical Sciences (code number: IR.SBMU.PHNM.1395.450) and Ahvaz Jundishapur University of Medical Sciences (code number: IR.AJUMS.REC.1395.730). This study was conducted in the outpatient clinic of the hemoglobinopathy department of a university hospital affiliated to Ahvaz University of Medical Sciences, during April to September 2019.

### Hypotheses

There are four hypotheses in the study:

1. The level of the SOC of SCD patients is the predictor of their physical HRQoL
2. The level of the SOC of SCD patients is a predictor of their mental HRQoL
3. The level of the SE of SCD patients is a predictor of their physical HRQoL
4. The level of the SE of SCD patients is a predictor of their mental HRQoL

### Sampling and data collection

All eligible and registered patients with SCD diagnosis at the outpatient clinic of the hemoglobinopathy department of the university hospital were invited to participate in the study. Through consecutive sampling during a 6-month period, among 113 patients in daily visits, 97 patients were entered into the study. From this sample, 14 patients either did not return the questionnaires or failed to complete them, so the final sample consisted of 83 patients. Patients over 16 years old with a diagnosis of SCD or sickle thalassemia in their medical records were eligible in this study (inclusion criteria). At the time of sampling, pain crisis episode in patients was considered as exclusion criteria. All participants were informed about the aims of the study and signed the informed consent form. Data was collected through four self-reported instruments by a trained registered nurse at the hospital**.**

### Measurements

#### Demographic-Clinical Information Questionnaire

This questionnaire included variables of age, sex, marital status, job status, education, SCD genotype, hydroxyurea consumption, arginine consumption, history of blood transfusion, and iron-chelating therapy. According to results of clinical variables in previous studies, comorbidities and/or chronic conditions are added to the questionnaire [5, 6], including splenectomy, heart, renal, bone or joint diseases, and acute chest syndrome**.**

#### Short-Form Health Survey SF-36 (RAND 36-item)

This is one of the most commonly used generic questionnaires of HRQoL measurement which was developed in the RAND Medical Outcomes Study during the 1980s [28]. The RAND 36-item comprising of 36 items with eight subscales: physical functioning (10 items), physical role functions (4 items), bodily pain (2 items), general health (5 items), social functioning (2 items), emotional role functioning (3 items), vitality (4 items), and mental health (5 items) [29, 30]. Four subscales of “physical functioning,” “physical role functioning,” “bodily pain,” and “general health” construct “Physical Component Summary” (PCS). Moreover, four subscales of “social functioning,” “emotional role functioning,” “vitality,” and “mental health” construct “Mental Component Summary” (MCS). Scores of the various parts of the questionnaire are transformed into the range of 0 to 100, the higher score represents, the better level of the HRQoL. A score of zero is equivalent to the worse HRQoL and a score of 100 is equivalent to the best HRQoL, and a score of 50 shows the average HRQoL [29, 30]. The validity and reliability of the Persian version of the RAND 36-item have been evaluated in Iran [29]. In this study, the reliability of the questionnaire and its subscales were satisfactory using Cronbach’s alpha coefficients in the range of 0.73–0.84.

#### Sickle Cell Self-Efficacy Scale (SCSES)

The SCSES was developed by Edwards et al. as a valid and reliable scale in 2000 and comprising of 9 items [31]. The patients’ responses were rated on a 5-point Likert scale ranging from 1 (not at all] to 5 (completely sure). The overall score of the scale varies from 9 to 45, which a higher score indicates better self-efficacy in SCD patients. The score of the scale is categorized into three levels of low SE (9–20.99), moderate SE (21–32.99), and high SE (33–45). This scale was translated and validated into the Persian language [32]. In the present study, the reliability of the SCSES was satisfactory using Cronbach’s alpha coefficient (0.80).

#### Sense of Coherence (SOC-13) Scale

The SOC of patients was measured using the short form of the SOC scale. It was developed by Antonovsky (1987) which consists of 13 items with three subscales: comprehensibility (5 items), manageability (4 items), and meaningfulness (4 items). The individuals’ responses are rated on a 7-point semantic scale ranging from 1 to 7. The total score ranges from 13 to 91 with a higher score, indicating a stronger SOC [24]. The validity and reliability of the Persian version of the SOC have been evaluated in Iran [33]. In this study, the reliability of the scale was satisfactory using Cronbach’s alpha coefficient (0.74).

### Data analysis

Data analysis was run using the SPSS version 20 (IBM Corporation, Armonk, NY, USA). Independent t-test and one-way ANOVA (analysis of variance) were used to compare the mean score of the PCS, the MCS, the SOC, and the SCSES in terms of dichotomous demographic and clinical variables. Significant demographic and clinical variables in addition to the SOC and the SCSES were entered into the regression models as independent variables. The relationship between outcome variables (the PCS, the MCS, the SOC, and the SCSES) was evaluated in a correlation matrix by Pearson correlation coefficients before running regression analyses. Two multiple linear regression analyses were conducted by stepwise method to determine the predictors of the physical and mental HRQoL in SCD patients.

## Results

### Descriptive results

The mean age of patients was 26.34 ± 8.19 years old. Most of them were single (63.9%), and female (55.4%). Demographic and clinical characteristics of the patients according to the mean levels of their HRQoL (the PCS and the MCS), the SOC, and the SCSES are presented in Table 1. It shows that there were no significant differences between categories of demographic and clinical variables in terms of the mean level of patients’ PCS, except for variables of marital status (*p* = 0.036) and blood transfusion (*p* = 0.011) as well as variables of blood transfusion (*p* = 0.005), hydroxyurea consumption (*p* = 0.004), and iron-chelating therapy (*p* = 0.041) in terms of the mean level of the MCS. No significant differences were found between categories of demographic and clinical characteristics in terms of the mean level of the SOC and the SCSES, except for education level (*p* < 0.05) (Table 1).

**Table 1:** Demographic and clinical variables of SCD patients based on four outcome variables; the PCS, the MCS, the SOC, and the SCSES (*n* = 83)

Variables		Number (%)	PCS (M ± SD)	MCS (M ± SD)	SCSES (M ± SD)	Total SOC (M ± SD)
Age	< 25	46(55.4)	41.53 ± 18.43	49.34 ± 18.07	25.08 ± 6.43	50.56 ± 15.53
	≤ 26	37(44.6)	39.39 ± 15.65	51.80 ± 17.95	28.05 ± 7.32	54.70 ± 15.02
	*p* value		0.575	0.537	0.053	0.225
Sex	Male	37(44.6)	42.36 ± 15.00	54.63 ± 18.02	26.59 ± 6.67	55.27 ± 14.45
	Female	46(55.4)	39.14 ± 18.78	47.07 ± 17.35	26.26 ± 7.26	50.10 ± 15.82
	*p* value		0.399	0.056	0.830	0.129
Marital status	Single	56(67.5)	43.31 ± 17.99	51.06 ± 17.13	26.48 ± 6.90	51.05 ± 15.84
	Married	27(32.5)	34.90 ± 14.01	49.16 ± 19.80	26.25 ± 7.20	55.22 ± 14.15
	*p* value		0.036	0.654	0.892	0.249
Job status	Unemployed	44(54.0)	39.70 ± 16.21	47.53 ± 14.98	27.43 ± 7.82	51.02 ± 15.89
	Employed	14(16.9)	39.95 ± 15.03	55.10 ± 20.21	26.78 ± 6.75	51.42 ± 13.61
	Student	25(24.1)	42.47 ± 20.24	52.94 ± 21.04	24.40 ± 5.02	55.40 ± 15.46
	*p* value		0.807	0.278	0.217	0.511
Education	< College	66(79.5)	41.60 ± 17.76	49.12 ± 17.26	25.62 ± 6.31	50.57 ± 15.36
	≥ College	17(20.5)	36.58 ± 14.49	55.56 ± 20.12	29.47 ± 8.61	59.52 ± 13.45
	*p* value		0.285	0.189	0.041	0.031
Sickle cell disease genotype	HbSS^*^	62(74.7)	41.63 ± 15.86	50.23 ± 17.86	27.14 ± 6.64	52.88 ± 15.47
	S-ß-Thal ^**^	21(25.3)	37.47 ± 20.70	51.04 ± 18.62	24.23 ± 7.58	51.00 ± 15.27
	*p* value		0.340	0.859	0.099	0.629
Blood transfusion	No	16(19.3)	50.31 ± 19.16	61.65 ± 16.72	26.43 ± 7.64	53.31 ± 12.04
	Yes	67(80.7)	38.25 ± 15.96	47.76 ± 17.29	26.40 ± 6.85	52.19 ± 16.11
	*p* value		0.011	0.005	0.986	0.795
Arginine consumption	No	72 (86.7)	41.92 ± 17.25	50.46 ± 18.00	25.93 ± 6.98	53.01 ± 14.98
	Yes	11 (13.3)	31.76 ± 14.46	50.29 ± 18.45	29.54 ± 6.23	48.45 ± 17.87
	*p* value		0.067	0.977	0.109	0.362
Hydroxyurea consumption	No	35(42.2)	40.19 ± 16.97	56.95 ± 16.23	26.11 ± 8.24	53.85 ± 16.86
	Yes	48(57.8)	40.85 ± 17.50	45.69 ± 17.80	26.62 ± 5.94	51.35 ± 14.24
	*p* value		0.863	0.004	0.744	0.467
Iron-chelating therapy	No	72(86.7)	40.33 ± 17.21	48.87 ± 18.27	26.15 ± 6.97	51.52 ± 15.83
	Yes	11(13.3)	42.15 ± 17.73	60.71 ± 11.56	28.09 ± 7.00	58.18 ± 10.58
	*p* value		0.746	0.041	0.393	0.182
Splenectomy	No	61(73.5)	41.61 ± 16.82	50.45 ± 18.41	26.88 ± 6.09	52.62 ± 15.65
	Yes	22(26.5)	37.69 ± 18.23	50.41 ± 17.02	25.09 ± 7.11	51.81 ± 14.83
	*p* value		0.362	0.994	0.303	0.835
Bone and joint disease	No	44(53)	41.74 ± 16.93	50.13 ± 18.57	26.13 ± 6.45	54.22 ± 15.59
	Yes	39(47)	39.26 ± 17.57	50.78 ± 17.45	26.71 ± 7.57	50.35 ± 15.02
	*p* value		0.514	0.871	0.707	0.254
Heart disease	No	77(92.8)	41.07 ± 17.40	50.49 ± 17.27	26.05 ± 6.77	52.53 ± 15.11
	Yes	6(7.2)	34.16 ± 13.49	49.72 ± 27.36	31 ± 8.43	50.83 ± 19.74
	*p* value		0.346	0.920	0.094	0.796
Renal disease	No	77(92.8)	40.34 ± 17.5	50.36 ± 17.94	26.28 ± 7.14	52.87 ± 15.4
	Yes	6(7.2)	43.54 ± 13.12	51.48 ± 19.74	28.00 ± 4.04	46.5 ± 14.72
	*p* value		0.664	0.884	0.565	0.331
Acute chest syndrome	No	27(32.5)	41.13 ± 21.37	52.79 ± 18.17	28.18 ± 8.71	53.44 ± 13.58
	Yes	56(67.5)	40.31 ± 14.96	49.30 ± 17.89	25.55 ± 5.84	51.91 ± 16.23
	*p* value		0.840	0.410	0.107	0.673

*SCD*, Sickle cell disease; *PCS*, Physical Component Summary; *MCS*, Mental Component Summary; *SCSES*, Sickle Cell Self-Efficacy Scale; *SOC*, Sense of Coherence

*Sickle Cell Anemia

**Sickle Beta Thalassemia

The mean scores of the RAND 36-item (eight subscales and two summaries), the SOC scale with three subscales, and the SCSES in SCD patients are shown in Table 2. The mean scores of the PCS, the MCS, the SOC and the SCSES were 40.57 ± 17.18 (range: 0–100), 50.44 ± 17.95 (range: 0–100), 52.40 ± 15.35 (range: 13–91), and 26.40 ± 6.96 (range: 9–45), respectively. Moreover, 61.4% and 19.3% of patients reported moderate and high SE, respectively. However, 19.3% of patients reported a low level of SE in this study (not shown in the Table).

**Table 2:** Descriptive data for outcome variables, including the RAND SF-36, SOC, and the SCSES in SCD patients (*n* = 83)

Outcome variables	Mean ± SD	Range in the scales
RAND SF-36		
Physical functioning	53.01** ± **23.59	0–100
Role limitations due to physical health	28.55** ± **24.69	0–100
Role limitations due to emotional problems	42.60** ± **38.35	0–100
Energy/fatigue	49.27** ± **20.63	0–100
Emotional well-being	55.18** ± **22.83	0–100
Social functioning	54.70** ± **23.43	0–100
Pain	37.62** ± **29.39	0–100
General health	46.98** ± **20.27	0––100
PCS	40.57** ± **17.18	0–100
MCS	50.44** ± **17.95	0–100
Total SOC	52.40** ± **15.35	13–91
Meaningfulness	17.65** ± **6.20	4–28
Manageability	15.28** ± **5.74	4–28
Comprehensibility	19.46** ± **6.56	5–35
SCSES	26.40** ± **6.96	9–45

*SCD*, sickle cell disease; *PCS*, Physical Component Summary; *MCS*, Mental Component Summary; *SCSES*, Sickle Cell Self-Efficacy Scale; *SOC*, sense of coherence

### Regression analyses results

As a prerequisite of regression analyses, a correlation matrix was done. A significant positive correlation was found between the SCSES with the PCS (*r* = 0.30, *P* < 0.006), and the MCS (*r* = 0.22, *P* < 0.04). Also, a significant positive correlation was established between the SOC with the MCS (*r* = 0.42, *P* < 0.001), and the SCSES (*r* = 0.22, *P* = 0.03) (Table 3).

**Table 3:** Correlation matrix between variables of the PCS, the MSC, the SOC, and the SCSES in SCD patients

Variables	SOC	PCS	MCS	SCSES
SOC	1			
PCS	0.05	1		
MCS	0.42**	0.40**	1	
SCSES	0.22*	0.30**	0.22*	1

According to Pearson correlation coefficients: *p < 0.05; **p < 0.001

*SCD*, Sickle cell disease; *PCS*, Physical Component Summary; *MCS*, Mental Component Summary; *SCSES*, Sickle Cell Self-Efficacy Scale; *SOC*, Sense of Coherence

Two multiple linear regression analyses with a stepwise method were run to determine the predictors of physical and mental HRQoL in SCD patients (Table 4). The results showed that hypotheses numbers two and three were accepted [the level of the SOC of SCD patients is the predictor of their mental HRQoL (*β* = 0.367, *p* < 0.001); the level of the SE of SCD patients is a predictor of their physical HRQoL (*β* = 0.301, *p* = 0.004)]. But, hypotheses numbers one and four were rejected [the level of the SOC of SCD patients is the predictor of their physical HRQoL (*β* =  − 0.024, *p* = 0.819); the level of the SE of SCD patients is a predictor of their mental HRQoL (*β* = 0.133, *p* = 0.159)]. The level of the SOC together with blood transfusion history, iron-chelating therapy, and hydroxyurea consumption in patients predicted 32% of the variances in the MCS. The level of the SE and blood transfusion history together in patients predicted 14% of the variances in the PCS (Table 4).

**Table 4:** Summary results of the multiple linear regression analyses with the PCS and the MCS as dependent variables

Dependent variables	Summary model	Predictors (Independent variables)	B	SE	β	t	*p*
PCS	R = 0.41	Constant	30.66	7.742	-	3.96	<0.001
	R^2^ = 0.16						
	Adj. *R*^2^ = 0.14	Self-efficacy	0.74	0.25	0.301	2.95	0.004
	*P* = 0.001	Blood transfusion	–12.03	4.41	–0.278	–2.72	0.008
MCS	R = 0.59	Constant	41.54	7.01	-	5.92	< 0.001
	R^2^ = 0.35	SOC	0.42	0.10	0.367	3.97	< 0.001
	Adj. *R*^2^ = 0.32	Blood transfusion	–13.35	4.30	–0.295	–3.10	0.003
	*P* < 0.001	Iron-chelating therapy	10.91	4.97	0.207	2.19	0.031
		Hydroxyurea	–7.31	3.39	–0.202	–2.15	0.034

*PCS* Physical Component summary, *MCS* Mental Component Summary

## Discussion

The aim of this cross-sectional study was to determine the level of the physical and mental HRQoL, the SOC, and the SE in a sample of SCD patients, and to explore the predictor role of the SOC and/or SE for patients’ physical and mental HRQoL. First, our results showed that the level of physical and mental HRQoL of patients was poor and average, respectively. However, their physical HRQoL was worse than their mental. It is not a surprising result as we can find the same results in earlier studies. SCD is a chronic disease and impacts on different dimensions of HRQoL in SCD patients compared to the healthy peers [1, 34–36]. But an interesting result in our study is related to blood transfusion history in SCD patients. Our patients with a history of blood transfusion showed a poor mental and physical HRQoL, respectively compared to those patients who have no history. This is consistent with earlier studies [37–39] which reported impaired physical and mental HRQoL in SCD patients with a blood transfusion history. In contrast, several recent studies showed different results. They reported better HRQoL and lower complications in SCD patients with a blood transfusion history [40–42]. Evidence shows that red blood cell transfusion can reduce many complications and risks, such as hemoglobin S concentration, pain, acute coronary syndrome, and stroke in patients [9]. However, frequent or chronic blood transfusion can bring about adverse effects in patients like iron overload and accumulation in the vital organs resulting in serious tissue damage [43]. Therefore, further research is suggested using valid and reliable scales in patients receiving short- or long-term RBC transfusions [44].

Moreover, our patients who took Iron-chelating therapy experienced better mental HRQoL compared to those patients who did not. Our result is parallel with a study which found that adolescents’ quality of life with β-thalassemia significantly improved using oral deferasirox [45]. Senol et al. [44] in an observational study evaluated SCD patients’ quality of life and their satisfaction with receiving DFX chelation therapy. They found a contrary result and the chelation therapy caused a negative impact on the patients’ quality of life, which probably shows patients’ poor compliance to the therapy. Poor compliance can be referred to as side effects of the chelating medicines which include abdominal pain, nausea, vomiting, and changes in renal and liver function. Goulas et al. [46] explained in their study that the quality of life of patients receiving chelation therapy is dependent on the type of iron chelation treatment they receive.

It is also remarkable that our patients who got hydroxyurea experienced lower mental HRQoL compared to those patients who did not. Despite documented benefits of hydroxyurea for pediatrics and young adults with SCD [47, 48], Ceveher et al. [49] proposed that quality of life and compliance of SCD patients might be low due to not sufficiently effective hydroxyurea therapy, comorbidities, concomitant medication use, and side effects. This issue can be explained in part by the previous patients' perceptions of treatment benefits versus risks, and their disease severity. Patients are more willing to accept the risk of hydroxyurea side effects if they had more severe forms of the SCD [43]. But patients with more hospitalization history perceived hydroxyurea as a less beneficial medicine [44].

Furthermore, we found a striking result for SOC which was measured for the first time in SCD patients in our context. We found no similar studies with the concept of the SOC in SCD patients. Also in our context, there is a lack of normative data for SOC in the population, therefore we compared our results with other patients. In our study, the mean level of the SOC in SCD patients was lower than the patients with different chronic diseases in other studies [50–52]. This difference can be justified by the distinct pathogenesis of the SCD compared to other chronic diseases. SCD is a genetic disorder with the most severe clinical and hematologic features which accompanies the patient from birth to death [53]. SOC is a global orientation to life and a personal way of thinking, being, and acting with an inner trust, which reflects a person’s view of life and capacity to respond to stressful situations. It leads people to identify, benefit, use and reuse the resources at their disposal in a way that promotes their health and well-being. SOC can predict an individual’s health and quality of life from childhood to adulthood [25]. A lower level of the SOC in SCD patients shows that they need more attention and planning for health programs.

In the present study, the majority of patients reported moderate SE. This finding is consistent with the results of several studies [14, 15, 20, 54]. SE is considered as a main feature in individuals’ coping process and HRQoL [20]. It points to one’s personal assessment of his/her capacity to self-care, contribute to symptom management, and support proper functioning about chronic medical needs [21]. Also, it has been linked to improved health outcomes among SCD patients [20, 54]. As increasing individuals’ SE can potentially improve their self-care, it is suggested to develop specific health programs by focusing on increasing SE in SCD patients [15, 54].

Our regression models showed that patients with higher levels of the SOC reported better mental HRQoL but not physical. In other words, the level of the SOC showed to be the predictor of the mental HRQoL in SCD patients. A strong SOC is associated with a better perception of mental health and quality of life than the physical dimension [55]. Our result is supported by the findings of a study by Galletta et al. [56]. They stated that SOC is a psychological process that mostly impacts patients’ mental health status [56]. Also, Rohani et al. [50] in part of their study results reported the mediating role of the SOC for mental HRQoL from pre-diagnosis of breast cancer to 6-month later. The role of the SOC in the context of our study assists in a better perception of how a higher SOC performs as a psychological factor in improving SCD patients’ HRQoL. Having a better knowledge of a patient’s SOC and how it affects their HRQoL may assist to identify patients who are at a greater risk of maladaptation with the SCD trajectory and help clinicians and nurses to improve health education interventions in direction of strengthening patients’ SOC [57].

Moreover, our results showed that the level of SE in SCD patients was the predictor of their physical HRQoL but not their mental. It means that patients with a higher SE reported better physical HRQoL. This result is consistent with the level of SE in patients with other chronic diseases. These studies have also shown that SE is positively related to physical HRQoL and can explain significant changes in it [58, 59]. However, in an earlier study in the same context in Iran, the predictive role of the SE was found for both dimensions of HRQoL; physical and mental. However, in that study, SE was a stronger predictor for the mental HRQoL in comparison to the physical [14]. It can be argued that the more number of the sample in that study might influence the study results. However, if we compare two predictors of HRQoL in the present study, our findings showed that the SOC was the predictor of the mental HRQoL, and the SE was for the physical HRQoL. Although the correlation between the two variables of the SOC and the SE was not high, but the hypothesis of the mediating role of the SOC can be proposed in the relationships of these variables with HRQoL. To find a new perspective, testing the reciprocal relationships of these variables and mediating the role of the SOC in future research with larger sample size is suggested**.**

### Strengths and limitations of the study

Measurement of the SOC in the study is one of the strengths of our study. To the authors’ knowledge, this is the first study that measured the level of the SOC among SCD patients and compared it with earlier studies in patients with other chronic diseases. This study has several limitations. First, although the number of participants was as high as to do analysis, selection of participants from one university hospital limits the generalizability of the findings. Secondly, we could not study the causal relationships among the study variables. Therefore, prospective studies are suggested to examine the long-term relationships between the variables and testing the mediating role of the SOC. Finally, response bias could be happening during responding to the self-reported questionnaires by participants.

## Conclusions

Based on our results, SCD patients reported their physical and mental HRQoL in the level of poor and average, respectively. Patients who received a blood transfusion and hydroxyurea experienced lower HRQoL. However, patients with Iron-chelating therapy reported improved HRQoL. Also, our results show that SOC was the predictor of the mental HRQoL, but SE was the predictor of the physical HRQoL. These results can assist to clinicians and nurses to plan holistic clinical interventions by focusing on the SOC and the SE to improve SCD patients’ HRQoL.
